# Conversion from electrocardiosignals to equivalent electrical sources on heart surface

**DOI:** 10.1186/s12859-020-3354-8

**Published:** 2020-03-11

**Authors:** G. V. Zhikhareva, Mikhail N. Kramm, O. N. Bodin, Ralf Seepold, Natividad Martinez Madrid, A. I. Chernikov, Y. A. Kupriyanova, N. A. Zhuravleva

**Affiliations:** 1 National Research University “MPEI”, Moscow, Russia; 2 Penza State University, Penza, Russia; 3 HTWG Konstanz, Ubiquitous Computing Lab, Alfred-Wachtel-Str. 8, Konstanz, 78462 Germany; 4 I.M. Sechenov First Moscow State Medical University, Institute of Digital Medicine, 2-4, Bolshaya Pirogovskaya st., Moscow, 119435 Russia; 5 Reutlingen University, IoTLab Internet of Things, Alteburgstr. 150, Reutlingen, 72762 Germany

**Keywords:** Multichannel, Electrocardiographic leads, Torso, Heart, Electric potential, Reconstruction, Equivalent electric sources, Maps of distributions, Cellular automata

## Abstract

**Background:**

The actual task of electrocardiographic examinations is to increase the reliability of diagnosing the condition of the heart. Within the framework of this task, an important direction is the solution of the inverse problem of electrocardiography, based on the processing of electrocardiographic signals of multichannel cardio leads at known electrode coordinates in these leads (Titomir et al. Noninvasiv electrocardiotopography, 2003), (Macfarlane et al. Comprehensive Electrocardiology, 2nd ed. (Chapter 9), 2011).

**Results:**

In order to obtain more detailed information about the electrical activity of the heart, we carry out a reconstruction of the distribution of equivalent electrical sources on the heart surface. In this area, we hold reconstruction of the equivalent sources during the cardiac cycle at relatively low hardware cost. ECG maps of electrical potentials on the surface of the torso (TSPM) and electrical sources on the surface of the heart (HSSM) were studied for different times of the cardiac cycle. We carried out a visual and quantitative comparison of these maps in the presence of pathological regions of different localization. For this purpose we used the model of the heart electrical activity, based on cellular automata.

**Conclusions:**

The model of cellular automata allows us to consider the processes of heart excitation in the presence of pathological regions of various sizes and localization. It is shown, that changes in the distribution of electrical sources on the surface of the epicardium in the presence of pathological areas with disturbances in the conduction of heart excitation are much more noticeable than changes in ECG maps on the torso surface.

## Background

The electrocardiographic method of heart investigation is one of the most common in cardiology. It is important to extract additional information on the electrical activity of the heart, using data from multichannel electrocardiographic leads, that include both recorded electrocardiosignals (ECS) and the coordinates of the electrodes placed on the surface of the human torso [1]. Since the potential distribution in a conducting medium is described by the Poisson equation, this leads to attenuation of the spatial harmonics in the potential map on the surface of the torso (TSPM) and, therefore, leads to loses of small details in the image. It means, that the electric potential, recorded with some electrode, is an integral characteristic of bioelectric activity. It is difficult to link individual TSPM area with specific area of the heart epicardial surface. In this connection, we are interested in solution of the inverse problem of electrocardiography, i. e. reconstruction of equivalent electric sources on heart surface, based on the records of multichannel electrocardiographic leads.

The inverse problem of electrocardiography is ill-posed, i.e. small errors in the measured signals or in geometric models can lead to errors and instabilities in the solution. Note the known methods for solving this problem: Tikhonov’s regularization method [2–4], truncated singular value decomposition (TSVD) method [5, 6] and some close approaches [4]. Statistical approaches include: Bayesian maximum a posteriori (MAP) estimation, when solution is the potential distribution that maximizes the posterior probability density function [7, 8]. Among the Statistical approaches we can mention Minimum relative entropy method (MRE), based on inferring a probability density function from a set of constraints and prior information [9]. However, for the Bayesian MAP approach it is necessary to know the a priori expected means of potentials on the epicardium and covariance matrix. Concerning the MRE method, it requires setting a priori parameters: the lower and upper bounds, the expected means of potentials, and the expected uncertainty in the model. In practice, the performance of TSVD regularization is often indistinguishable from that of Tikhonov regularization [10]: despite the different conceptual basis for the two approaches, the resulting equations are closely connected. Since the main features of the distribution of epicardial potential in these approaches are reasonably reconstructed, we use the method of Tikhonov’s regularization below [11].

We are interested in the reconstruction of an equivalent electric heart generator in the form of a simple layers of electrical sources (SLS) and double layers of electrical sources (DLS) on the surface of the epicardium [1, 11, 12]. As a result of such reconstruction, it is possible to receive spatio-temporal maps of equivalent sources for analysis of the heart electrical activity. Reconstruction algorithms for distributions of simple layer sources (SLS) density have been proposed and approved on simulated and real electrocardiosignals [13, 14]. One of the objectives of this article is to analyze the results of the reconstruction of the distributions of both SLS and DLS density.

The second essential issue of this article is the confirmation of the relevance of the reconstruction of the distribution of equivalent sources for the diagnosis of disturbances in the conduction of heart excitation. For this purpose we compare electrical sources distribution maps on the heart surface (HSSM) with potential distribution maps on the torso surface (TSPM). We consider the possibility of detecting the changes in HSSM in the presence of pathological areas with disturbances in heart excitation. This is especially important for the early diagnosis of myocardial conduction disturbances, when pathological changes in the distribution of potentials on the torso surface are still not noticeable.

For this purpose we use the model of cellular automata [15], which allows simulation of the heart electrical activity, taking into account the features of autowave processes in pathological areas [16–18]. We got, that in a number of localizations of pathological regions, TSPM does not allow us to detect this areas, while the HSSM allows us not only to detect, but also to estimate the sizes of these areas.

We indicate the dynamics of the heart electrical activity by the space-time mapping of equivalent electrical sources in HSSM.

## Methods

### Inverse problem

Relation between surface distributions of electrical sources and the measured electric potential in cases of simple and double sources layers follows from Poisson’s equation:

1 $$\begin{array}{@{}rcl@{}} \phi(\bar{r}) = \frac{1}{4 \pi \sigma} \int_{S} \frac{\gamma_{S} (\bar{r})}{|\bar{r}-\bar{r}'|}dS;  \\\\ \phi(\bar{r}) = \frac{1}{4 \pi \sigma} \int_{S} D_{S}(\bar{r}) \frac{\partial}{\partial n} \frac{1}{|\bar{r}-\bar{r}'|}dS, \end{array} $$

where *γ*_*S*_ – density of unipolar moment, *D*_*S*_ – density of dipolar moment, $|\bar {r}-\bar {r}'|$ – distance from a torso surface point (radius-vector $\bar {r}$) to the integration point (radius-vector $\bar {r}'$) on epicardium surface S, *∂*/*∂n* – a derivative in the direction of a normal to this surface.

The task is reduced to the decision of systems of the linear algebraic equations:

2 $$\begin{array}{@{}rcl@{}}  \phi_{k} = \sum_{l} A_{\gamma k l} \gamma_{Sl}; \enspace \enspace \phi_{k} = \sum_{l} A_{D k l}D_{Sl}, \end{array} $$

with matrixes of coefficients:

3 $$\begin{array}{@{}rcl@{}} A_{\gamma k l} = \frac{1}{4 \pi \sigma} \frac{\Delta S_{l}}{|\bar{r_{k}}-\bar{r_{l}}'|}; \enspace  \\\\ A_{D k l} = \frac{1}{4 \pi \sigma} \frac{\Delta S_{l} cos \alpha_{l k}}{|\bar{r_{k}}-\bar{r_{l}}'|^{2}},  \end{array} $$

where *ΔS*_*l*_ – the square of a surface epicardium element with number *l*, *α*_*lk*_ – angle between a vector of the dipolar moment *D*_*Sl*_ and a vector $\bar {r_{k}}-\bar {r_{l}}'$, linking surface elements with numbers *l* and *k* on epicardium surface and a torso.

The decision of systems of the Eq. (2) belongs to incorrect tasks, therefore the method of A.N. Tikhonov regularization was applied to find the normal decision [2]: we need to search of a vector *z*^*α*^, minimizing functional:

4 $$\begin{array}{@{}rcl@{}}  M^{\alpha} [z, \phi] = ||Az - \phi||^{2} + \alpha_{a} ||z||^{2}, \enspace \alpha_{a} > 0, \end{array} $$

with regularization parameter *α*_*a*_, defined on discrepancy, i.e. from a condition ||*Az*^*α*^−−*ϕ*||=*δ*_1_, where *δ*_1_ – total mean square deviation of the measured potentials from one’s, calculated by means of the found decision *z*^*α*^=*γ*_*S*_ or *z*^*α*^=*D*_*S*_. To find the minimum of the functional (4) it is necessary to solve system of the linear algebraic equations:

5 $$\begin{array}{@{}rcl@{}}  \alpha_{a} z^{\alpha}_{k} + \sum_{j} C_{kj} z^{\alpha}_{k}= b_{k}, \end{array} $$

with

6 $$\begin{array}{@{}rcl@{}}  C_{kj}=\sum_{i} A_{ik} A_{ij}; \qquad b_{k}=\sum_{i} A_{ik} \phi_{i}. \end{array} $$

Calculations using formulas (5) and (6) give a solution to the inverse problem in cases of a simple or double layer of sources on the epicardium using matrices *A*_*γkl*_ or *A*_*Dkl*_ in (2) respectively.

### Interpolation of torso surface and the surface potential map

**Fig. 1:**
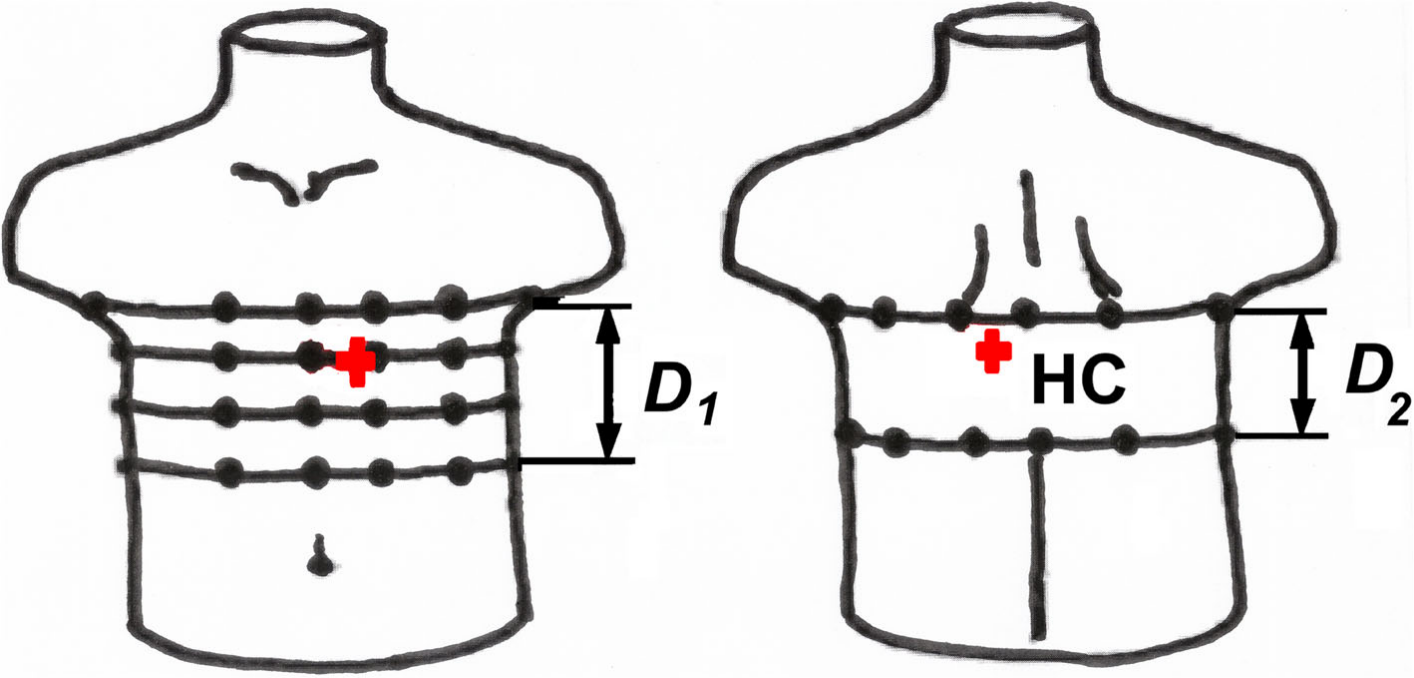
System of electrode leads: **a** chest; **b** back; “+” – corresponds to heart center

Electric potentials were registered by means of the multielectrode measuring system shown in Fig. 1 [13]. Electrodes were installed on elastic belts placed on the torso. This system is suitable for both men and women with small breasts. For women with large breasts the position of the electrodes on the front of the torso can be adjusted. In accordance with [11, 13], the number of points with a known potential on the torso and the number of sampling points of the sources density on the epicardium should be about 10^3^ in order to satisfactorily describe the spatial spectrum of the density distribution of electrical sources on the epicardium. So, using 1500 points, the spatial step on the surface of the epicardium is about 5 mm. Since our multielectrode system allows to measure the electric potential at 40–50 points on the torso, it is necessary to carry out the interpolation of the electric potential on a fine grid of points on the torso surface. In the first step we determined the coordinates of the base points on the torso surface (about 10^2^ points), including the coordinates of the electrodes. For this we used a special program that processes digital torso photos with base points. In the second step we passed from the base points to a fine grid of points on the surface of the torso and interpolated the electric potential on this resulting grid of points.

### Simulation of the heart electrical activity

When developing, approbating and researching the diagnostic significance of algorithms for solving the inverse problem of electrocardiography, multi-electrode ECS are needed, as well as accurate information about the sources of these signals: size, location in the chest, the presence or absence of pathological changes. But there are practically no such bases for real multielectrode ECS. Therefore, in order to obtain multielectrode ECS, simulation of the heart electrical activity is important.

**Fig. 2:**
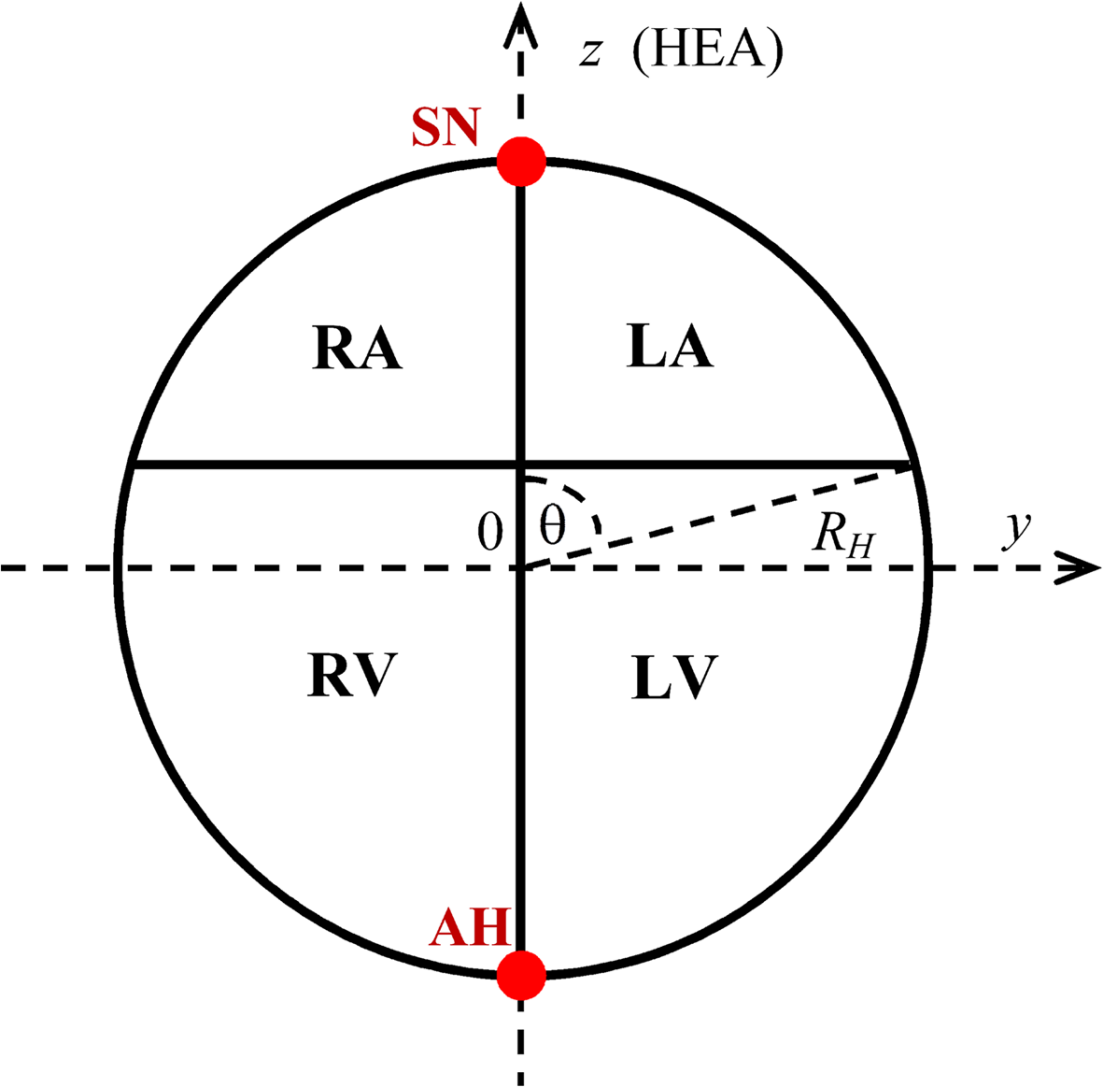
Geometric model of quasi-epicardium; SN – sinus node; AH – apex of the heart; HEA – electric axis of the heart

The theory of cellular automata (CA) [15] was used for simulation the dynamics of myocardial excitation. The heart is represented by a spherical quasi-epicardial surface with radius *R*_*H*_=4.6 cm, with a hexagonal grid applied to it dividing the surface on the CA [16]. The border of the atria and ventricles is determined by the angle *θ*=75^0^, the sinus node (SN) corresponds to the angle *θ*=0^0^, for the apex of the heart (AH) *θ*=180^0^ (Fig. 2). The plane *xz* in the coordinate system of the heart divides the heart into the left and right parts.

**Fig. 3:**
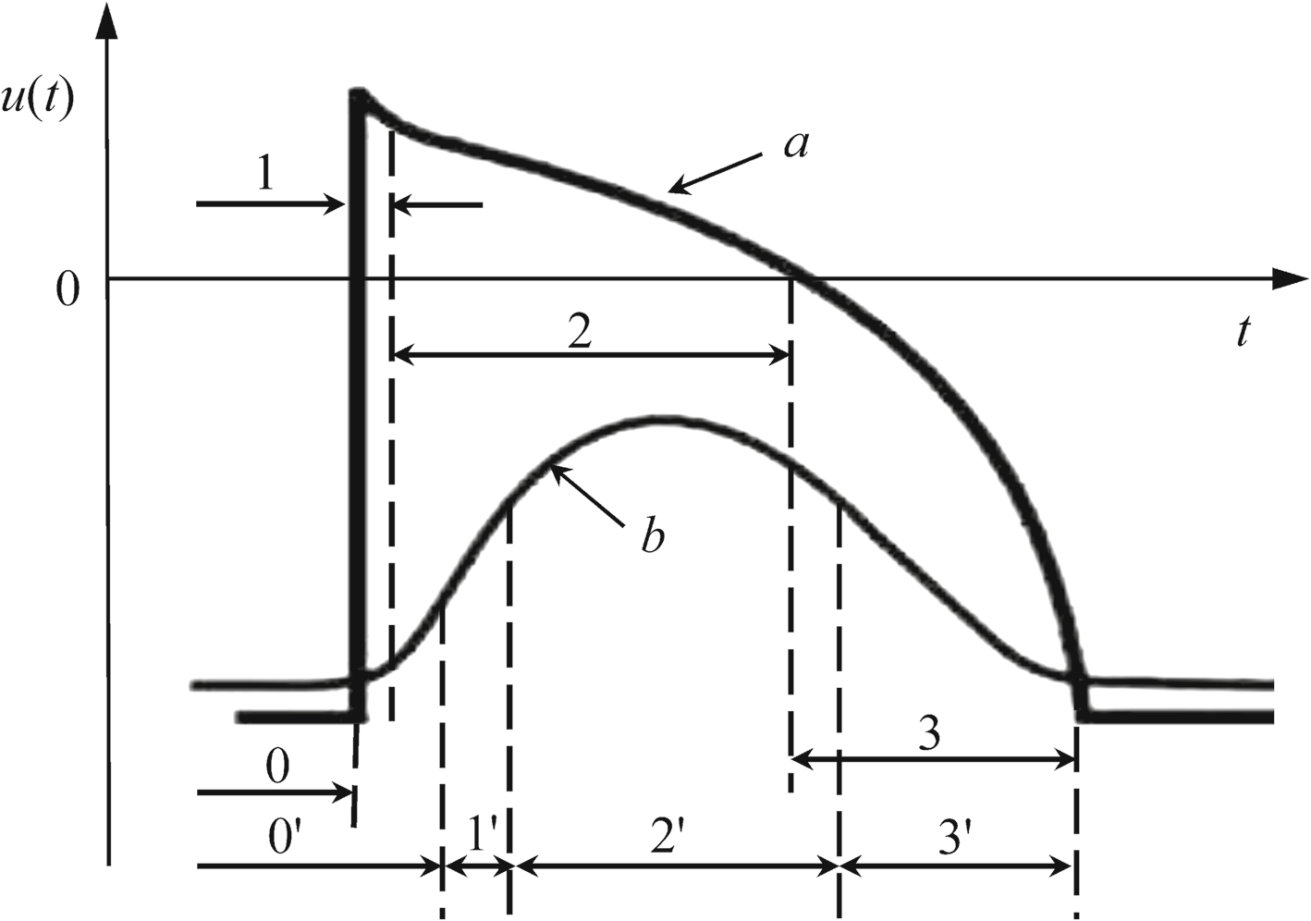
Transmembrane action potentials of myocardial cells (a – normal, b – in pathology) and the corresponding states of cellular automaton: 0, 0’ – rest; 1, 1’ – depolarization; 2, 2’ – refractoriness; 3, 3’ – repolarization

The rules of CA state change are determined, based on the phases of transmembrane potential of myocardial cells: 0 - rest, 1 – depolarization, 2 - refractoriness, 3 - repolarization (Fig. 3). The state 1 CA is transferred only from the state 0 if there is at least one CA in its neighborhood in the state 1. Then all the states sequentially replace each other, regardless of the states of neighboring cells.

Autowave processes are triggered by the drivers of the rhythm in the sinus node and in the apex of the heart: CA, located in the sinus node, at the zero moment of time (the beginning of the P wave) goes to the state 1, activating the Atria. Through 0.13s CA, located at the apex of the heart, goes into state 1, activating the ventricles.

With pathological changes in the myocardium we understand the appearance of areas with delayed excitation. Such changes are typical in some forms of ischemic myocardial damage in place of which may develop, subsequently, myocardial infarction [19]. In this case the action potential becomes "slow", which leads to a delay in switching cellular automaton extends phase 0’, i.e, the CA switches to state 1’ with a delay, state 1’ lasts much longer, also change the durations of phases 2’ and 3’ [17] (Fig. 3).

**Fig. 4:**
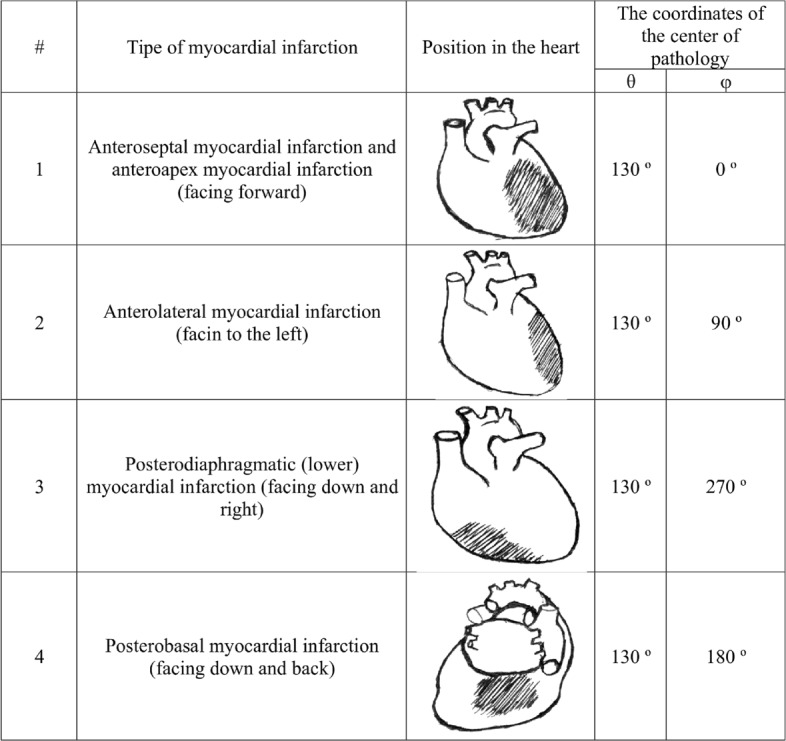
The coordinates of the centers of pathologies for various localizations in the heart generator model

The zones of location of pathological areas were selected for the localization of the most common types of myocardial infarction [20]. Spherical coordinates of the centers of the pathological areas on the surface of the heart generator are presented in Fig. 4; the angle *φ* was measured from the x-axis (Fig. 2). The sizes of the areas varied between 1 - 5 cm.

The process of calculating the potentials created by the heart generator is reduced to the following stages [18]:

1. Calculation of the matrix of modules of dipole moments of cellular automaton $\{D_{CA_{ij}}\}$ in discrete moments of time of a single cardiac cycle; *i* – number of cellular automaton, *j* – number of the time point of a single cardiac cycle. The algorithm of calculation of the CA dipole moments $\{D_{CA_{ij}}\}$, which is in phases 1 or 3 (i.e. when a significant change in the transmembrane potential and the active flow of membrane currents are observed), is based on the analysis of the electrical status of cell membranes of the myocardium in different parts of the heart [11, 17]. CA dipole moments in phases 0 and 2 (when membrane currents are small) are assumed to be zero.

2. The calculation of matrix potentials produced by each cellular automaton in interesting points of space, $\{\phi _{CA_{ik}}\}$; *i* is the number of CA, *k* – number of point space. The dipole moments of all cellular automaton are assumed to be equal to one (*D*_0_=1).

For a homogeneous conductive medium potentials are determined by the formula:

7 $$\begin{array}{@{}rcl@{}} \phi_{CA_{ik}} = \frac{(x_{k} - x_{CA_{i}})D_{0_{xi}}+(y_{k} - y_{CA_{i}})D_{0_{yi}}+(z_{k} - z_{CA_{i}})D_{0_{zi}}}{4 \pi \sigma \sqrt{(x_{k}-x_{CA_{}i})^{2}+(y_{k}-y_{CA_{}i})^{2}+(z_{k}-z_{CA_{}i})^{2}}^{3}}, \end{array} $$

where (*x*_*k*_,*y*_*k*_,*z*_*k*_) – the coordinates of a point of determination the potential, $(x_{CA_{i}}, y_{CA_{i}}, z_{CA_{i}})$ – the coordinates of the centers of cellular automaton, $(D_{0_{xi}}, D_{0_{yi}}, D_{0_{zi}})$ – projections of unit vectors located in the centers of cellular automatons and directed along the normal to the spherical surface of the generator, *σ*=0.22 Sm/m – specific conductivity of the medium – the average value of the specific conductivity of the chest tissue [1].

3. Calculation of the potentials of the heart generator:

8 $$\begin{array}{@{}rcl@{}} \phi = D_{CA}^{T} \phi_{CA}, \end{array} $$

where *ϕ* – the matrix of potentials {*ϕ*_*jk*_} for time point with number *j* in space point with number *k*; $D_{CA}^{T}$ – the transposed matrix of the modules of the dipole moments of the CA, reflecting the dynamics of autowave processes on the surface of the epicardium (stage 1); *ϕ*_*CA*_ – matrix of potentials created by cellular automata separately (stage 2).

## Results

### Results of reconstruction

**Fig. 5:**
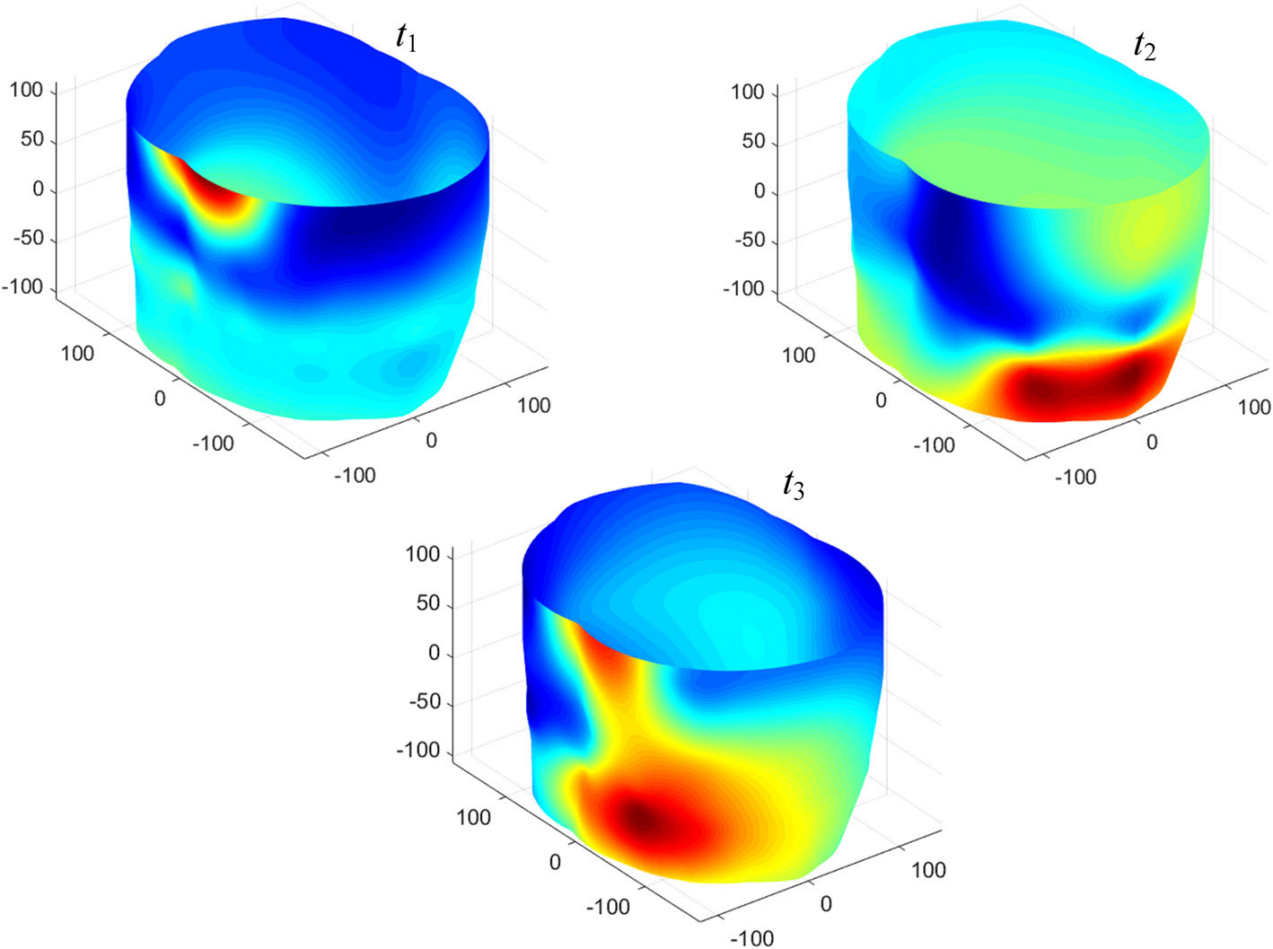
Torso surface potential maps for time points *t*_1_,*t*_2_ and *t*_3_ (Fig. 6); spatial coordinates are measured in millimeters

**Fig. 6:**
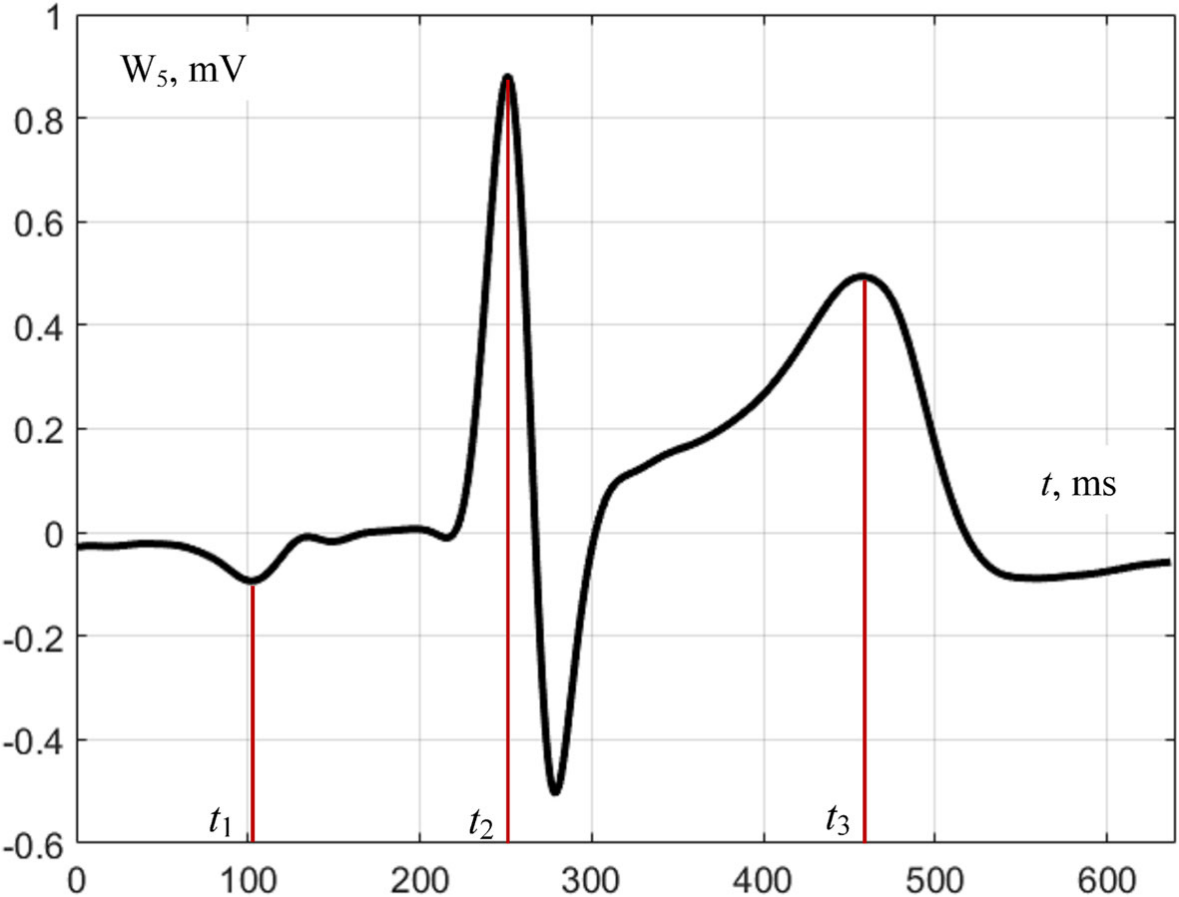
Time points of cardiocycle, the magnitude of the electrocardio signal is measured in millivolts

In Fig. 5 we presented torso surface potential maps (TSPM) for the time points, corresponding to P-, R- and T- waves of a cardiocycle (Fig. 6). Figures 5 and 6 are obtained for real electrocardiosignals. On Fig. 5 the magnitude of the electrical potential is displayed using the pseudocolor scale: the largest positive potential value is displayed by the largest saturation of red color, and for the negative potential – by the saturation of blue color. To reduce the noises level up to 1–2 *μV*, we used synchronously accumulated recordings of the cardiocycles in combination with digital filtration.

The presented methods were used for reconstruction of equivalent electrical sources on spherical quasi-epicardium, based on real multichannel records of electrocardiosignals. In order to exclude the effect of chest movements in the process of respiration on the results of the reconstruction of source distributions, we considered cardiocycles corresponding to the expiratory phase. For this purpose the shape of the respiratory wave in the cardiac rhythmogram was taken into account.

**Fig. 7:**
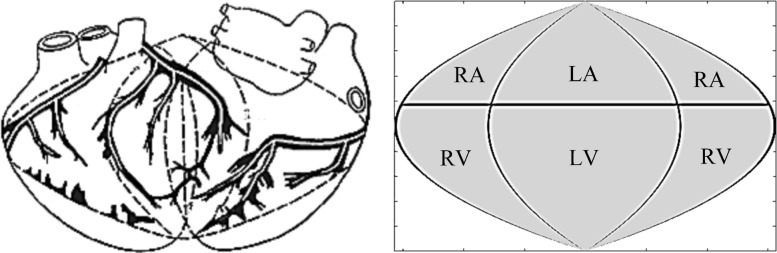
The plane projection of quasi-epicardium surface; areas of auricles and ventricles. RA and LA – the right and left atriums, RV and LV – the right and left ventricles

**Fig. 8:**
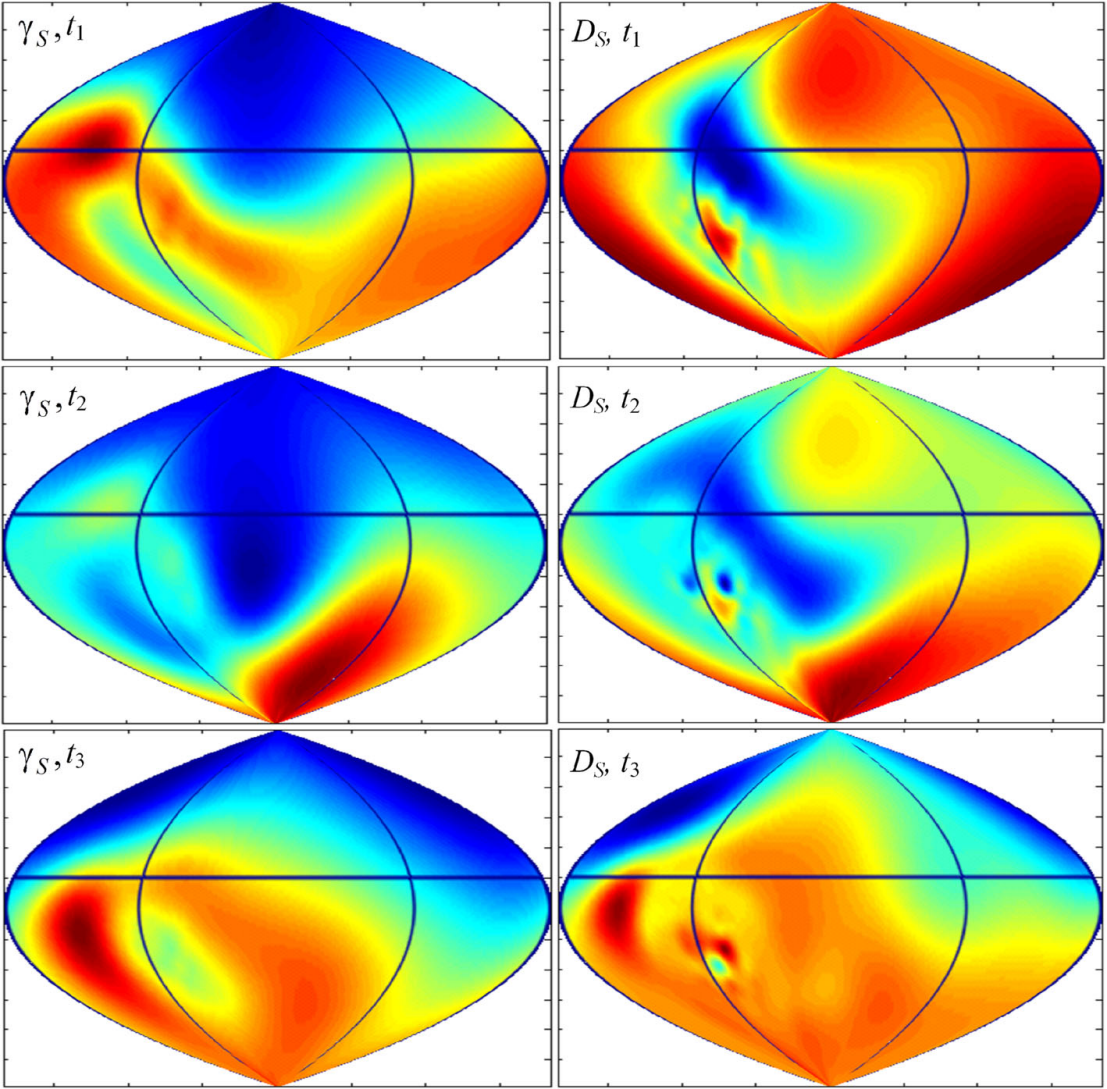
Heart surface source distributions SLS (*γ*_*S*_) and DLS (*D*_*S*_) for the time points, corresponding to tops of P, R and the T- waves of a cardiocycle (Fig. 6)

The received heart surface source distributions (HSSM) were represented on plane projection of the sphere quasi-epicardium (Fig. 7). In Fig. 8 we represent HSSMs for the time points, corresponding to tops of P, R and the T-waves of a cardiocycle (Fig. 6).

For convenience of visualization the scale of pseudo-colors on maps of Fig. 8 fits to different range of sources density. In accordance with Fig. 7, the central vertical line in Fig. 8 corresponds to the central regions of the left atrium and the left ventricle.

The source distributions for the cases of a simple and a double layer correspond to each other. The density of a simple layer reflects the density of effluent currents, and the density of a double layer reflects the behavior of the transmembrane potential in depolarization and repolarization of the heart. We observe the smaller details in the distribution of the sources of the double layer.

For time point *t*_1_, corresponding to the top of the P-wave, the region of the beginning of heart depolarization is located in the area of the right atrium. For time point *t*_2_, corresponding to the top of the R-wave, the distributions of electrical sources *γ*_*S*_ and *D*_*S*_ in Fig. 8 reflect the dipole structure of the electric heart generator [1]: the maximum positive potential is located in the region of the apex of the left ventricle, and the minimum negative potential is in the region of the left atrium.

Comparing the distributions of SLS and DLS, it can be noted that the activation areas of effluent currents (*γ*_*S*_), generally speaking, are different from the activation areas of the transmembrane potential, characterized by the density of dipole sources *D*_*S*_. This difference is dependent on the relative location and level of the sources in these areas on the surface of the epicardium.

Thus, using space-time mapping, one can observe the movement of electrical activity zones directly on the heart surface during the cardiac cycle. The visualization of electrical activity, that we use with 2D plane projection of the sphere quasi-epicardium, is convenient, since it allows us to observe the behavior of electrical sources simultaneously at all points on the surface of the quasi-epicardium.

### Comparative analysis of potential distribution maps on the torso surface and of equivalent sources on the quasi-epicard

Using the CA heart generator model, TSPM were calculated for normal condition (*ϕ*) and for pathologies with different localization and size ($\tilde {\phi }$). The model of the chest in the form of an elliptical cylinder with the typical size and placement of the heart was used in the calculations of HSPM.

We also calculated the distributions of simple (*γ*_*S*_) and double (*D*_*S*_) layers of equivalent sources on quasi-epicardium (HSSM), including presence of pathologies ($\tilde {\gamma _{S}}$ and $\tilde {D_{S}}$).

To estimate the changes in distributions due to the presence of pathological regions, we calculated the distributions of difference $\Delta \phi = | \phi - \tilde {\phi } |$ and the total surface area, for which this difference is greater than 10% of the maximum value *max*{*Δϕ*}, determined for all surface points for all moments of time:

9 $$\begin{array}{@{}rcl@{}} S_{\phi j} = \sum_{k} \Delta S_{k}, \enspace \Delta \phi_{jk} > 0.1max\{\Delta\phi\}, \end{array} $$

where *j* – number of the time point, *k* – number of surface point, *ΔS*_*k*_ – square of the *k*-th element of the torso surface. Similarly, the analysis of distributions of equivalent sources on the quasi-epicardium was carried out. The results of the calculations are summarized in Table 1, here the limits of variation of characteristics correspond to the limits of the change in the pathological regions 1–5 cm; the area values of the pathological regions are also given as a percentage of the total square of the torso (for *max*{*S*_*ϕ*_}) or from the total square of the quasi-epicardium (for $max\{S_{\gamma _{S}}\}$ and $max\{S_{D_{S}}\}$).

**Table 1:** Maximum values of arrays $\protect \phantom {\dot {i}\!}S_{\phi }, S_{\gamma _{S}}, S_{D_{S}}$ at various localization and size of pathology

Localization of pathology	*max*{*S*_*ϕ*_},*cm*^2^(*%*)	$max\{S_{\gamma _{S}}\}, cm^{2} (\%)$	$max\{S_{D_{S}}\}, cm^{2} (\%)$
1	107.0−125.5*cm*^2^(2.3−2.7*%*)	1.0−8.0*cm*^2^(1.4−11.3*%*)	0.7−3.5*cm*^2^(1.0−5.0*%*)
2	1236.4−1251.2*cm*^2^(26.8−27.1*%*)	1.5−10.3*cm*^2^(2.1−14.5*%*)	0.7−5.7*cm*^2^(1.0−8.2*%*)
3	2557.7−2624.2*cm*^2^(55.4−56.9*%*)	1.5−10.3*cm*^2^(2.1−14.5*%*)	0.7−5.7*cm*^2^(1.0−8.2*%*)
4	2613.1−2757.0*cm*^2^(56.6−59.8*%*)	1.0−8.0*cm*^2^(1.4−11.3*%*)	0.7−3.5*cm*^2^(1.0−5.0*%*)

From Table 1 it follows, that the size of the regions of the quasi-epicardium, in which the *γ*_*S*_ and *D*_*S*_ distributions differ from the norm, is unit of *cm*^2^, which corresponds to the size of the pathological region.

A different picture is observed in the distribution of potentials on the surface of the torso. When the pathological region is turned to the anterior wall of the torso (type of localization 1), it is as close as possible to the potential measurement surface. In this case differences in the distribution of TSPM in normal and pathological conditions are concentrated in the region of the order of 100 *cm*^2^.

When the pathological region is turned to the left side, it is already more distant from the potential measurement surface, the area of differences in the distributions is "blurred": the area increases, practically by an order of magnitude, and the differences become weakly expressed.

When the pathological area is turned down, to the back wall of the torso and to the right side (localization types 3 and 4), it is as far from the potential measurement surface as possible. In this case, the effect of "erosion" is maximal: the area of differences in the TSPM is still doubled.

**Fig. 9:**
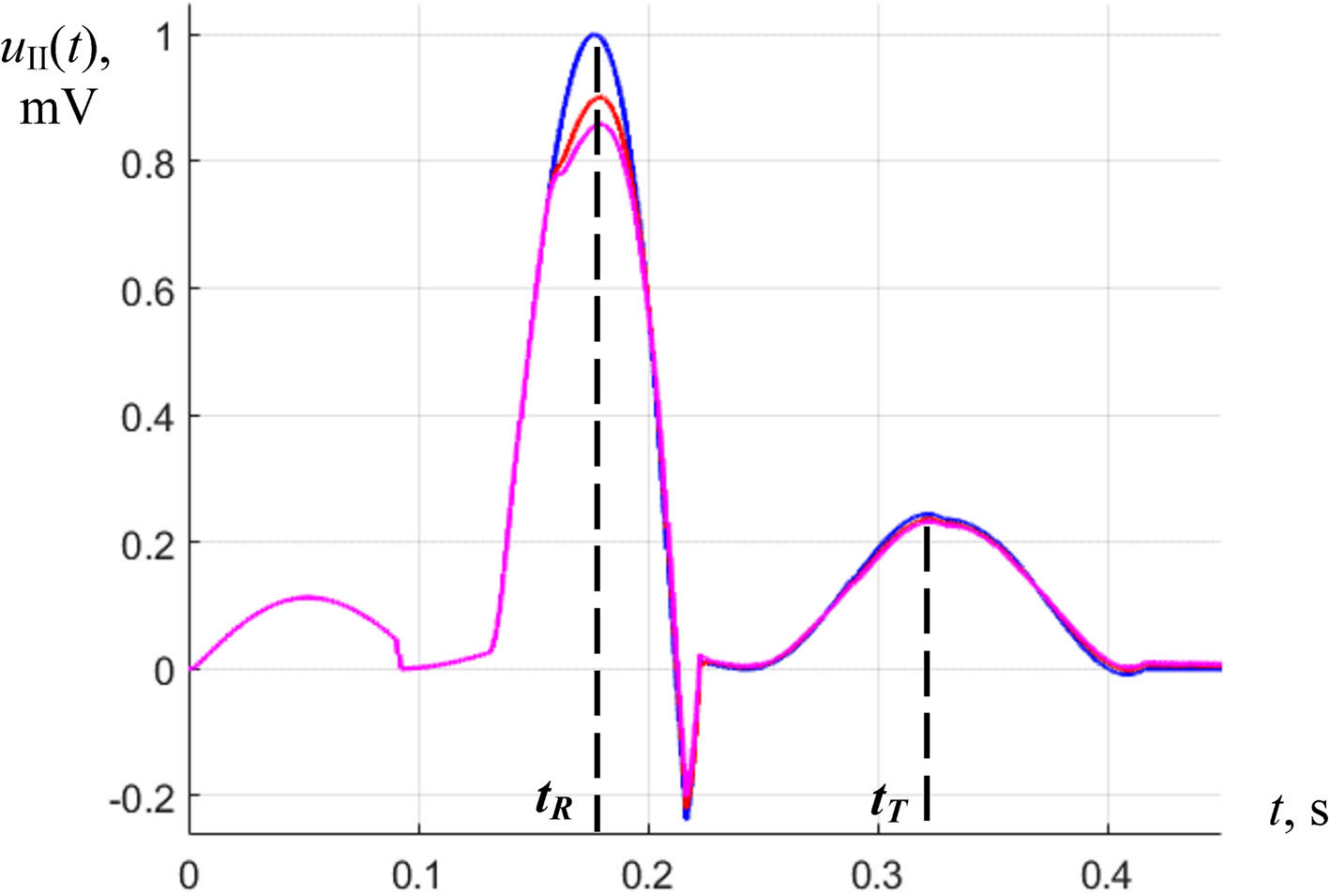
The ECG signal of the standard lead II: the blue line is normal, red line – in the presence of type 1 pathology, crimson line – in the presence of type 4 pathology

Let us consider in more detail the visualization of the modeling results. Figure 9 shows the result of the calculation of the ECS of the II standard lead in the norm and in the presence of pathology regions (3 cm in diameter) in anteroseptal (type 1) and posterobasal (type 4) localizations. On these signals, pathologies show a slight decrease in the R-wave amplitude. It should also be noted, that the ECS, shown in Fig. 9 by the blue line, corresponds in shape and amplitude to the real ECS in II standard lead; this is achieved due to the calibration of the cellular automaton model when choosing the model coefficients.

**Fig. 10:**
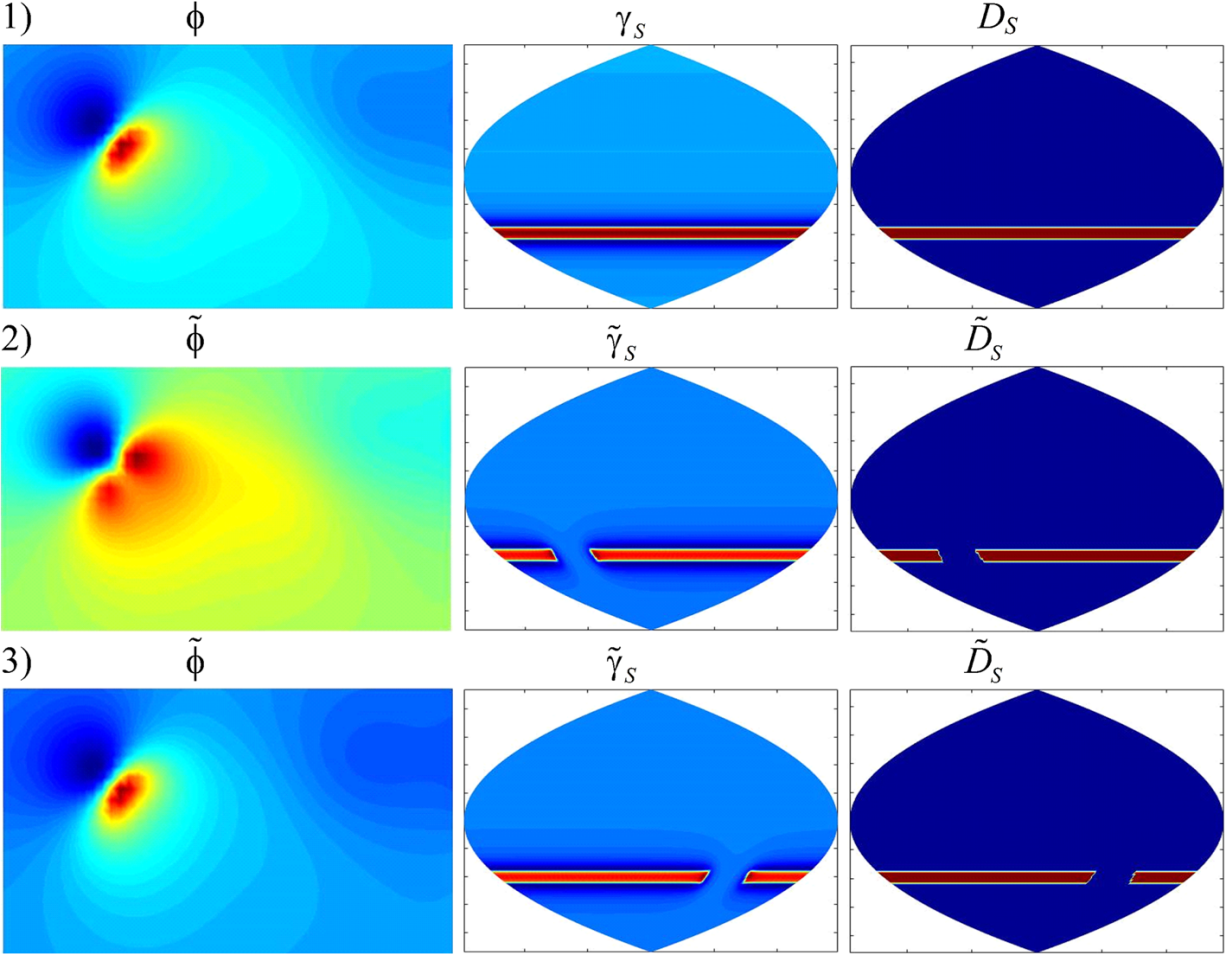
Distributions of potentials (*ϕ*) and equivalent sources (*γ*_*S*_ and *D*_*S*_) at the time point *t*_*R*_: **1** normal, **2** in the presence of type 1 pathology, **3** in the presence of type 4 pathology

**Fig. 11:**
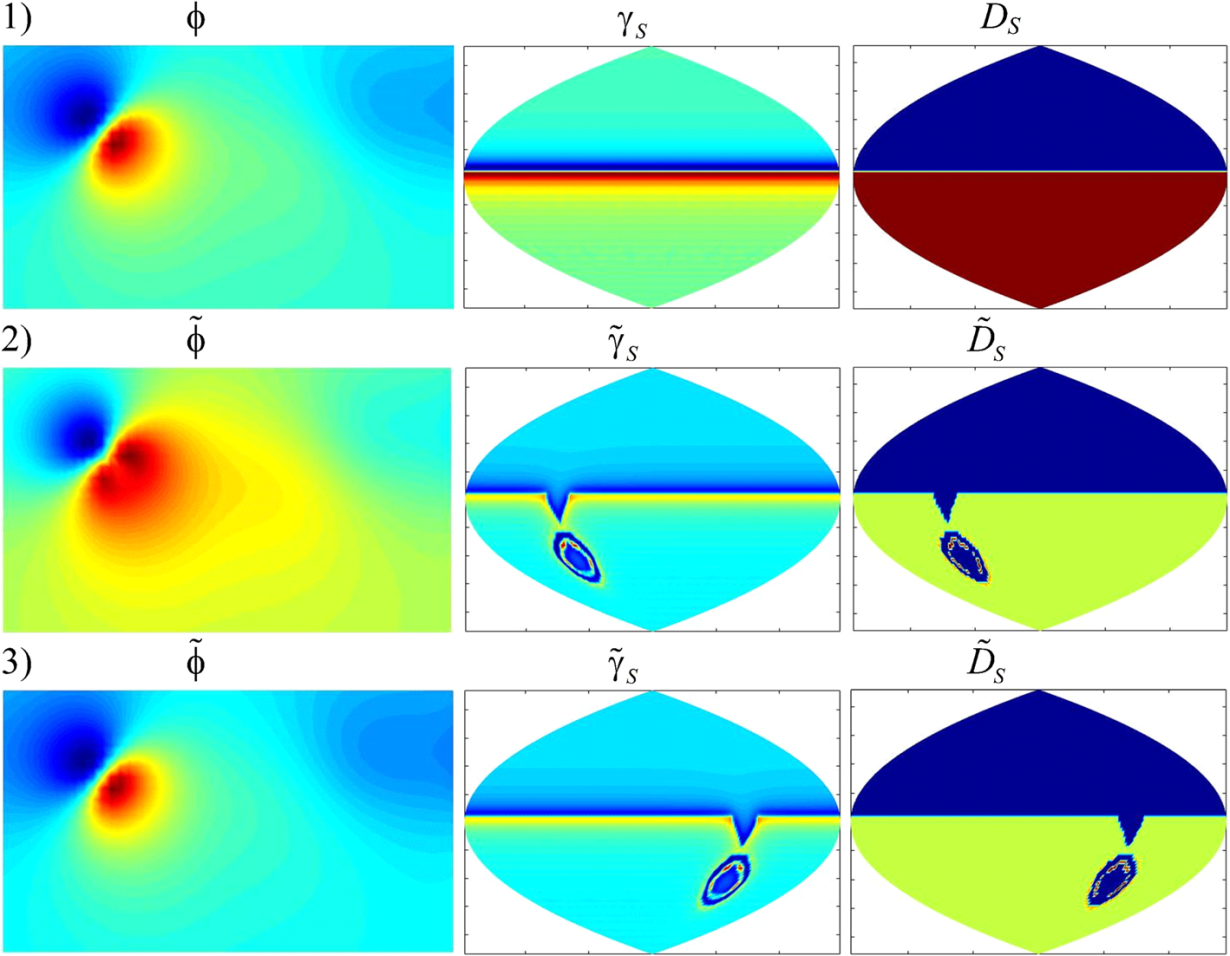
Distributions of potentials (*ϕ*) and equivalent sources (*γ*_*S*_ and *D*_*S*_) at the time point *t*_*T*_: **1** normal, **2** in the presence of type 1 pathology, **3** in the presence of type 4 pathology

Figures 10 and 11 show the analyzed distributions of potentials and equivalent sources at time points corresponding to the tops of R and T-waves (*t*_*R*_ and *t*_*T*_). At these times, waves of depolarization and repolarization of the ventricles pass through pathological regions.

At 1 type of localization of pathological area there are significant changes in the shape of the TSPM distributions (Figs. 10, 2 and 11, 2), that is, according to the TSPM, one can discover the presence of pathological changes in the myocardium and approximately determine its localization. With the 4 type of localization, in spite of the presence of a pathology of the same size, the changes in the distributions of the TSPM are not significant (Figs. 10, 3 and 11, 3), i.e. on TSPM it is impossible to discover neither the presence nor the localization of pathology.

Contrariwise, by distributions of equivalent sources on quasi-epicardium it is possible not only to determine the presence or absence of pathological changes, but also to discover their localization and even to estimate their sizes.

Thus, we have shown the importance of the transition from TSPMs to heart surface distributions of equivalent sources (HSSM), i.e. diagnostic value of using algorithms for reconstruction of equivalent source distributions on quasi-epicardium according to TSPM.

## Discussion

In this paper we addressed the issue of the relationship between the electrical potential distributions on the torso surface (TSPM) and the distributions of equivalent electrical sources on the heart surface (HSSM). Electrical potentials on the torso surface are recorded directly using the ECS of multichannel cardiac leads, therefore, in order to observe the TSPM, it is enough to conduct an ECS interpolation at known coordinates of electrodes on the torso surface. At the same time, to obtain HSSM, it is necessary to consider the inverse problem of electrocardiography and solve a system of linear algebraic equations (SLAE), connecting the electrical potential at each point of the torso surface grid with the source density values at all points of the heart surface grid. An additional problem here is the localization of the area of electrical sources, which is necessary to set the coordinates of the surface of the heart, taken into account in the SLAE coefficients. In an earlier paper [11] we proposed to localize this region based on the results of TSPM processing using the properties of the Laplace equation for the region between the torso surface and the heart surface. This approach is approximate, but it allows us to do without significant hardware costs associated with the use of a computer tomograph or MRI tomograph.

In the first part of this paper we showed the possibility of obtaining HSSM from the records of real multichannel ECSs. At the same time we used to show the distribution of electrical sources on a flat projection of a spherical quasi-epicard, surrounding the heart, which allows us to observe the electrical activity of the heart at once at all points on the heart surface.

In the second part of the article we examined the question of the expediency of transition from TSPM to HSSM, which is determined by the possibility of observing myocardial areas with pathologies of excitation. To do this, we conducted a mathematical simulation of the excitation in the heart muscle, using the model of cellular automata [16–18]. This approach allows us to calculate the electric potential in different phases of the cardiac cycle, including the moments of passage of the excitation front through the region with conduction pathology. We compared TSPM and HSSM with different sizes and locations of pathological areas. The main result is the ability to detect pathological areas of smaller size (about 1−8*cm*^2^) on HSSM maps as compared to TSPM maps, as well as determining the localization of these areas. The further development of this direction is related to the influence of the regularization coefficient upon receipt of HSSM by solving the SLAE on real ECS.

## Conclusion

We considered an algorithm for reconstructing the distribution maps of sources of heart electrical activity on the surface of the epicardium. An approach is based on the use of real multichannel ECS, received by the system of electrocardiographic leads and use of coordinates of the electrodes in these leads. The algorithm makes it possible to visualize maps of equivalent sources in the form of a simple (SLS) or double electric layer (DLS) for different times of the cardiac cycle. In contrast to the works [9, 10] our proposed application of distribution maps SLS and DLS allows us to compare the electrical heart activity in the form of behavior of both the transmembrane potential and the effluent epicardial currents. The visualization of electrical heart activity that we use with 2D plane projection of the sphere quasi-epicardium surface allows us to simultaneously observe state of activity at all points on the surface of the epicardium.

Note that for finding the position and size of the area of the epicardium, multichannel ECS are used directly and no additional equipment is required [11, 14]. For comparison, the approach described in [2, 3, 6] requires the results of a preliminary scan of the patient using CT or MRT.

The algorithm is focused on the study of conduction disturbances in the heart excitation.The paper analyzes the diagnostic capabilities of monitoring cardiac conduction disturbances. Using the model of cellular automata, we compared the distributions for TSPM and HSSM at different locations and different sizes of the region with pathological disturbances in the conduction of heart excitation (delayed myocardial excitation, typical in some forms of ischemic myocardial). We note, that pathological changes on the surface of the epicardium (HSSM) are significantly more noticeable than on the surface of the torso (TSPM).

We hope that the using of a volume model of the electrical heart activity with the help of cellular automata is promising in the future for improving the algorithms for solving the inverse problem of electrocardiography (IP ECG) in the direction of refining the parameters of these algorithms. So, it is useful to plan the use of this model to analyze the effect of internal torso inhomogeneities on the accuracy of the decision of the IP ECG, in the development of works [21–23]. In addition, it is promising to use the cellular automata model to clarify the a priori parameters the lower and upper bounds and the expected means of potentials in the Minimum relative entropy method [9].

## Data Availability

Model of the heart electrical activity, based on cellular automata, is available from previous publications [8,12].

## Abbreviations

AH: Apex of the heart
CA: Cellular automata
ECG: Electrocardiography
ECS: Electrocardiosignal
HEA: Electric axis of the heart
HSSM: Heart surface source map
SN: Sinus node
TSPM: Torso surface potential map
